# Arthroscopic Bone Block Procedure Using Iliac Crest Autograft and Labral Advancement and Repair

**DOI:** 10.1002/atn2.70156

**Published:** 2026-08-17

**Authors:** K. N. Subramanian, Aravind Ravichandran, Nirmal Raja, Jeash Narayan

**Affiliations:** ^1^ Vale Hospital Madurai India

## Abstract

Recurrent anterior shoulder instability with significant glenoid bone loss requires bony augmentation to restore stability and prevent recurrence. Arthroscopic iliac crest bone block reconstruction offers an anatomical solution while avoiding morbidity of coracoid transfer. This Technical Note outlines a simplified, reproducible method for preoperative planning, precise portal placement, complete labral mobilization, accurate graft contouring, controlled graft passage, and secure tensioned fixation with FiberTape (Arthrex, Naples, FL). Through this article, we have attempted to simplify the procedure and provide important technical tips to increase accuracy. The offered suggestions will be useful for beginners in learning this technique. This technique ensures proper graft fixation, natural tissue integration, and restoring the glenoid anatomy to the maximum in recurrent shoulder instability cases.

## Graphical abstract

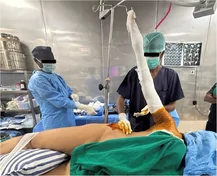

**VIDEO 1:** Arthroscopic bone block procedure using an iliac crest autograft with labral advancement and repair performed in a left shoulder, with the patient in the lateral decubitus position under traction. Arthroscopic visualization is obtained through the anteroinferior viewing portal, showing a large anterior glenoid defect with a disrupted labrum. The labrum is mobilized using a periosteal elevator from the 1 o'clock to the 6 o'clock position to achieve adequate liberation and excursion. Glenoid edge preparation is performed using radiofrequency ablation, followed by freshening of the raw bony glenoid margins with a curette and arthroscopic burr to create a bleeding bone bed. Sutures are passed through the liberated labrum using an antegrade suture‐passing device and lasso technique, retrieved through the trans‐subscapularis portal using a bird‐beak retriever, and parked. A glenoid drill guide is introduced through the posterior portal, and 2 drill holes are created in the anteroinferior glenoid. Guidewires are passed and exchanged for FiberTape sutures. Through the anteroinferior working portal, the iliac crest bone block is delivered using forceps and positioned flush against the anterior glenoid with gentle manipulation. The graft is tensioned to achieve firm compression against the glenoid surface, and fixation is secured. Using the previously parked sutures, the labrum is restored to its native footprint on the glenoid and stabilized with knotless anchors. A remplissage procedure is performed to address the engaging Hill‐Sachs lesion, resulting in restoration of the glenoid defect and labral anatomy with a well‐centered humeral head. Video content can be viewed at https://doi.org/10.1002/atn2.70156.

Glenohumeral instability, particularly recurrent anterior dislocations, often presents a complex challenge for orthopaedic surgeons. Soft tissue procedures, such as Bankart repair, work well in isolated labral lesions and glenoid bone defects that are less than <20%. Significant glenoid bone loss is a recognized risk factor for failure, necessitating resorting to bony procedures, increasing the glenoid surface area like the Latarjet and Eden‐Hybinette procedure.^1^ The traditional open Latarjet procedure, with coracoid transfer, has been viewed for years as the gold standard, with low rates of recurrence (approximately 2% to 5%). But it is associated with complications such as subscapularis dysfunction, and graft‐related issues.^2^ The arthroscopic Latarjet technique was introduced with the goals of reducing soft tissue trauma, improving graft placement accuracy, and potentially enhancing early postoperative recovery, yet this procedure carries a steep learning curve and similar complication rates to the open approach.^3^ More recently, arthroscopic iliac crest bone block procedures have become acceptable as an anatomical option that can preserve the coracoacromial arch and avoid coracoid harvest. Studies have documented high graft union rates (up to 100%), low recurrences (~6% to 7%), and a reliable restoration of the glenoid morphology.

Arthroscopic bone block procedure using the autologous iliac crest graft has become a viable and increasingly popular option for treating these bony deficiencies, with biological incorporation and restoration of native anatomy as its advantages while minimizing the morbidity associated with the open procedure. Though a relatively complex procedure, this technical note aims to break down the steps and outline a simplified approach to the arthroscopic bone block procedure utilizing an iliac crest graft for glenoid bone defects, emphasizing key steps for successful execution.

## SURGICAL TECHNIQUE

### Preoperative Planning

Patient assessment and thorough preoperative 3‐dimensional computed tomography (CT) imaging are of utmost importance in planning successfully for an arthroscopic bone block procedure with an iliac crest autograft. The planning is carried out through a detailed patient assessment and 3‐dimensional CT imaging.

The initial clinical evaluation involves obtaining the history concerning glenohumeral instability with details on the number and mechanism of dislocations along with any prior treatment. Furthermore, pain, range of motion, and stability are tested, along with associated injuries such as rotator cuff tears or Hill‐Sachs lesions. Age, activity level, and comorbidities such as smoking or diabetes are essential factors in evaluating healing potential and rehabilitation expectations. Patients then undergo CT scans with 3‐dimensional reconstruction.

CT imaging technique permits the accurate quantification of glenoid bone loss, with characterization in respect of defect area, percentage, and morphology. Moreover, it offers a detailed assessment of Hill‐Sachs lesions, determining their size, depth, and engagement, thereby allowing decisions regarding accompanying procedures such as remplissage.

A magnetic resonance imaging has an important and complementary role, being considered a superior method for the assessment of concomitant soft tissue injuries frequently seen in recurrent shoulder instability cases. It can confirm and characterize labral tears, assess rotator cuff tendons, and identify capsular laxity or articular cartilage damage.

### Patient Positioning

**FIGURE 1:**
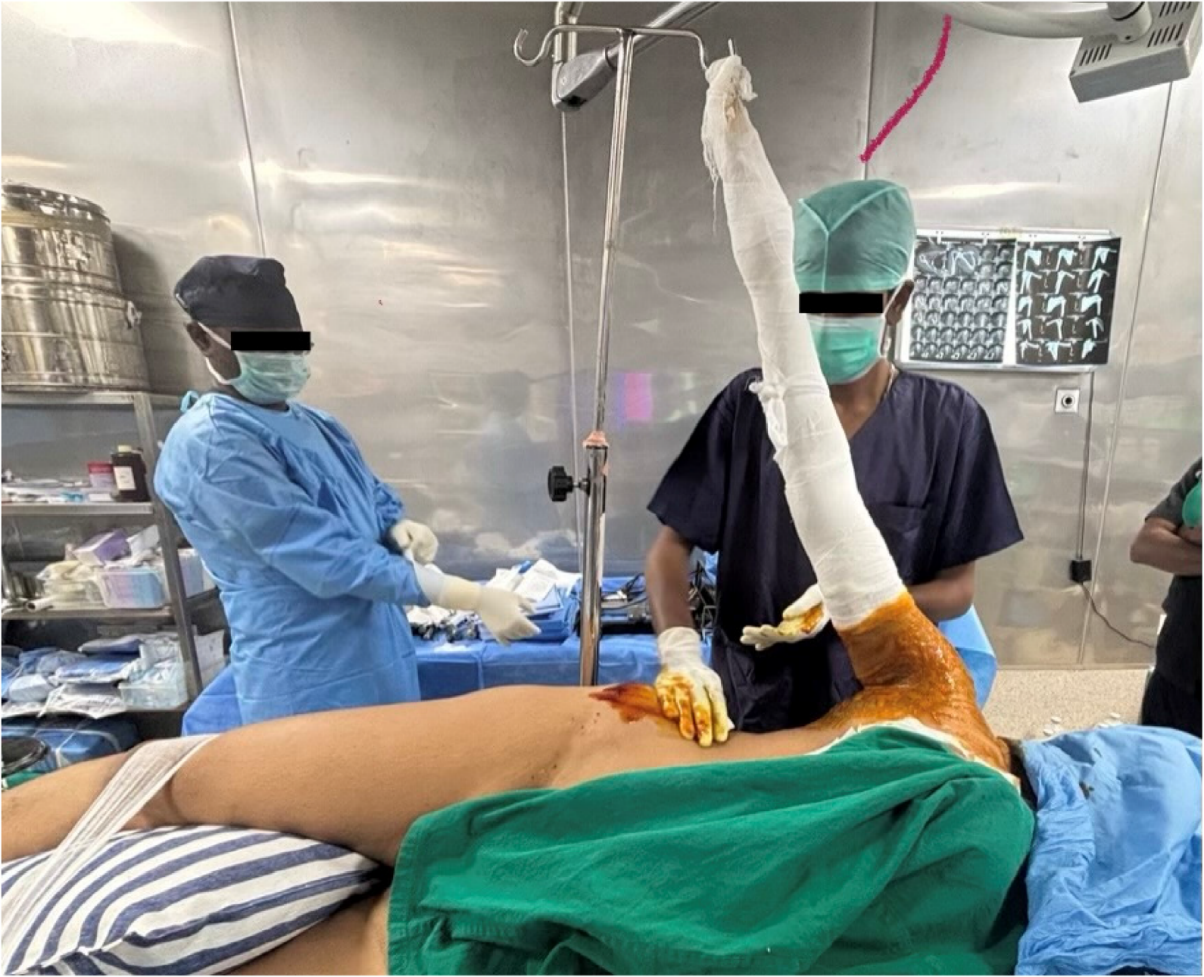
Patient positioning. The patient is placed in the left lateral position in traction with the operative shoulder and iliac crest area prepared and draped, allowing unrestricted access to the anterior shoulder, iliac crest graft harvest site, and optimal arthroscopic portal placement.

The patient is positioned in a lateral position with two bolsters on the same side: the proximal bolster placed below the inferior angle of the scapula and the distal bolster positioned at the sacral region. An additional bolster is placed on the opposite side, supporting the abdomen, with adequate padding to avoid excessive compression. The arm should be abducted to 70°, with the shoulder in a slightly floppy lateral position (Figure 1). This floppy lateral position with oblique tilt of pelvis helps in iliac crest bone graft harvesting.

### Graft Harvesting and Preparation

The harvesting step using the iliac crest autograft is vital for the success of the arthroscopic bone block procedure. The iliac crest has ample bone stock, is accessible, and has highly osteogenic, osteoconductive, and osteoinductive properties that allow good biological integration with the native glenoid.

After appropriate patient positioning and prepping of both the shoulder and the iliac crest areas, an approximately 3‐ to 5‐cm longitudinal skin incision is placed over the anterior part of the iliac crest, just 2 cm lateral to the anterior superior iliac spine. Caution is taken so as not to injure the lateral femoral cutaneous nerve. The dissection is then gently carried down to the periosteum.

**FIGURE 2:**
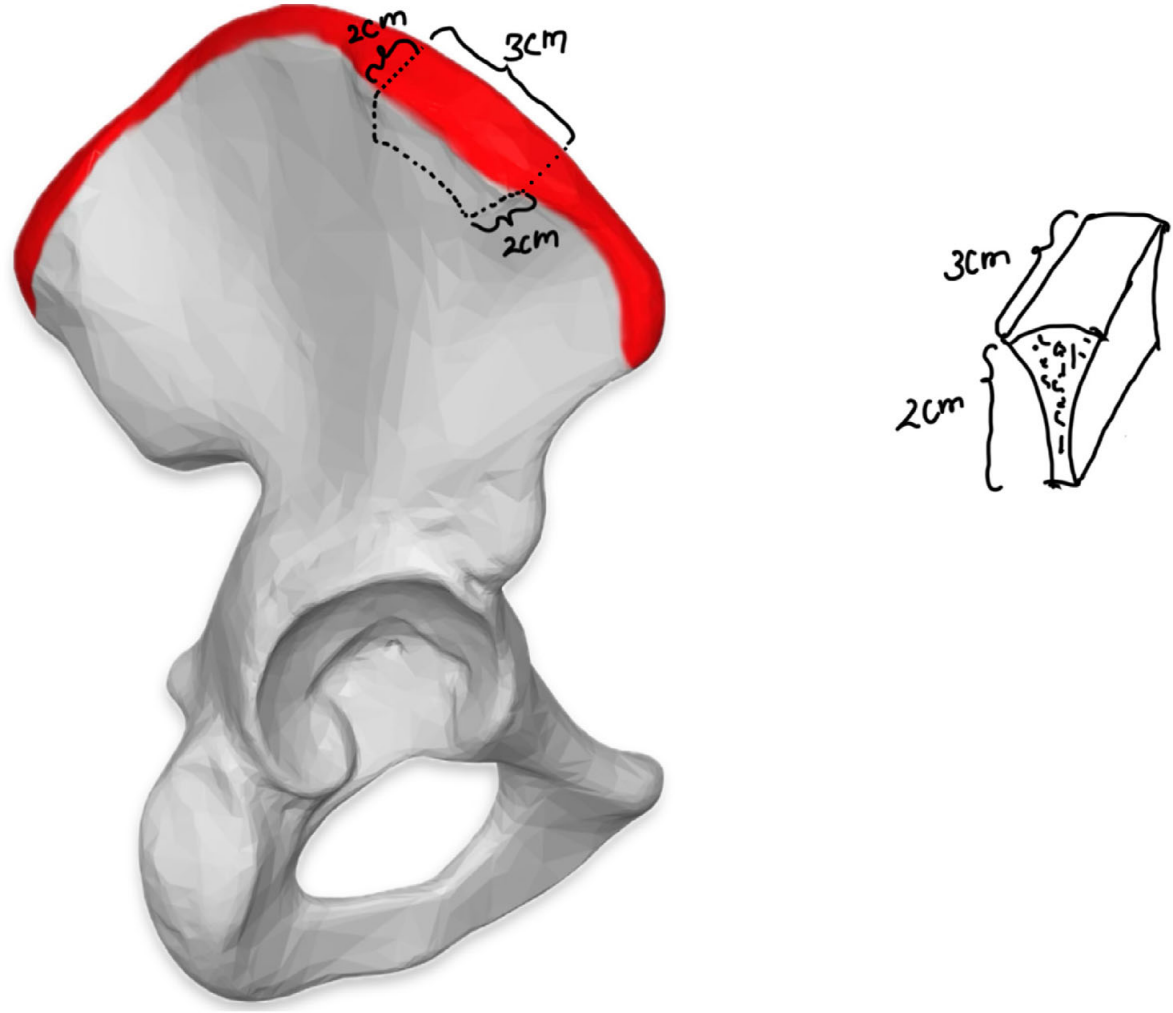
Iliac crest autograft harvested for use in the arthroscopic bone block procedure. The graft is obtained from anterior iliac crest of size 3 cm length 2 cm depth and width. The graft is sized appropriately for subsequent shaping and fixation.

A tricortical block is reconstructed, and the size of the graft is determined by preoperative CT planning of the glenoid defect, generally a graft size of roughly 3 cm in length, 2 cm in width, and 2 cm in thickness (Figure 2), with these measurements allowing for adequate reconstruction of the deficient glenoid surface while preserving the cortical integrity for mechanical strength.

**FIGURE 3:**
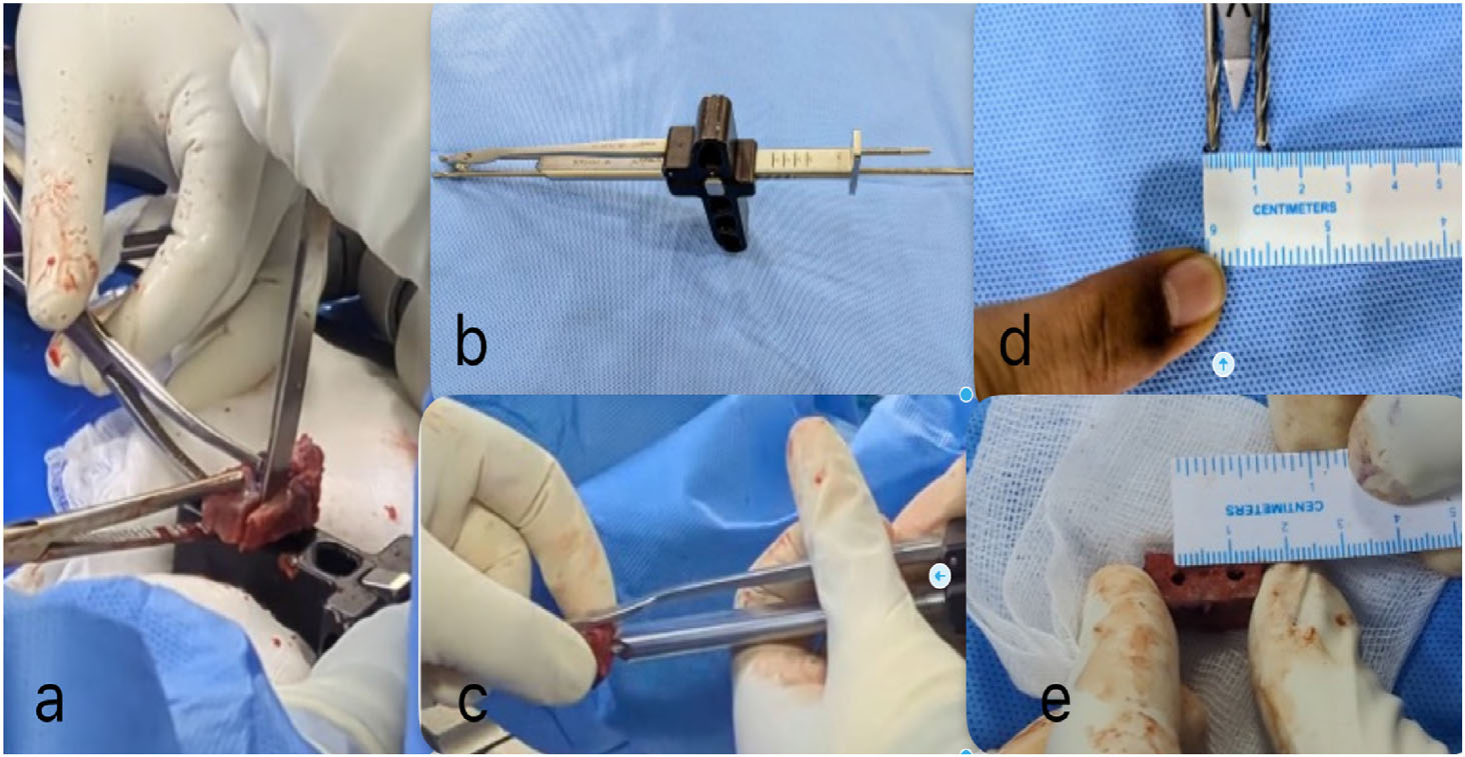
Stepwise preparation of the iliac crest bone graft. (a) Graft shaping using an osteotome and oscillating saw. (b) Arthrex (Naples, FL) bone graft jig applied to the graft for accurate drill hole placement. (c) Creation of drill holes through the jig. (d,e) Correspondence between the drill hole spacing in the jig and the prepared bone graft, ensuring reproducible graft positioning during fixation.

Once harvested, the graft is meticulously shaped on the back table using an oscillating saw and burrs to match the glenoid defect accurately (Figure 3a). Creating a flat articular surface that will intimately contact the prepared anterior glenoid edge is critical.

Two parallel holes are drilled through the graft in an anterior‐to‐posterior fashion (Figure 3c). Their positions correspond with those of the superior and inferior glenoid drill channels that will be prepared intra‐articularly. These holes will pass the FiberTape through for graft fixation and tensioning. Attention is given to confirm that the holes are centrally aligned and spaced adequately to allow even distribution of mechanical loads and to enable accurate graft positioning.

Once the final contouring and hole preparation are done, a thorough irrigation of the graft is undertaken using saline to remove all bone debris. The prepared graft is then covered in moist gauze and kept ready for implantation into the joint at the appropriate stage of the procedure.

### Portals and Diagnostic Arthroscopy

**FIGURE 4:**
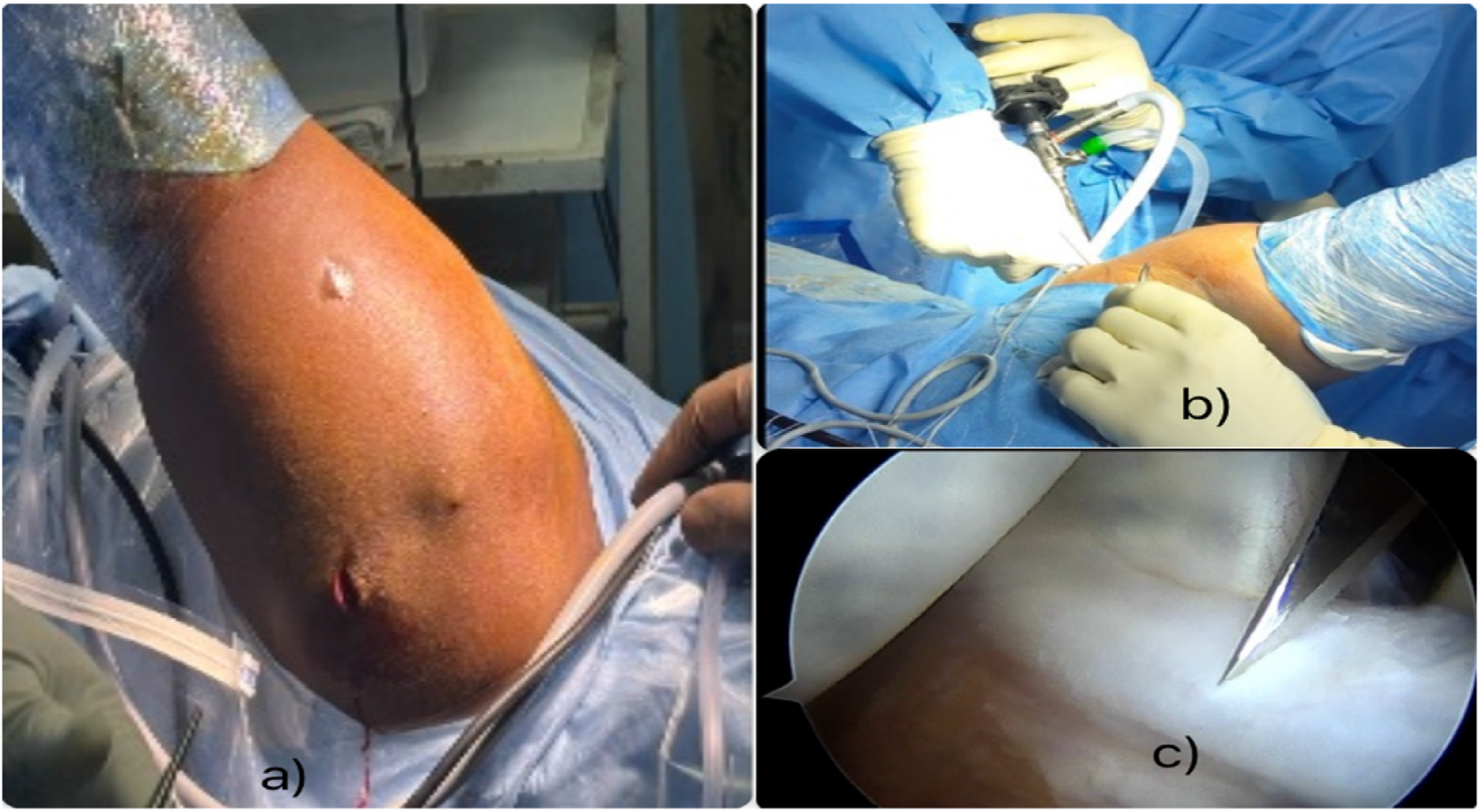
Arthroscopic portal creation for graft passage and fixation in a left shoulder. (a) Establishment of the standard posterior viewing portal; incision is made 2 cm inferior and medial to posterolateral border of acromion. (b) Creation of the anteroinferior working portal using an outside‐in technique, with the surgeon's index finger able to palpate the anterior glenoid to facilitate smooth graft passage. (c) Controlled enlargement of the anteroinferior portal using No. 11 scalpel blade to accommodate bone graft insertion.

A posterior portal is created with a stab skin incision using a No. 11 scalpel blade, extended vertically and perpendicular to the body plane (Figure 4a). The deep planes are dissected using fingers until the bone is reached. The humeral head, joint, and glenoid are palpated using the index finger. A trocar is then introduced into the joint by piercing the capsule, with the trocar directed toward the coracoid process.

Diagnostic shoulder arthroscopy is done (Video 1) visualizing the status of biceps, labrum, and the glenoid defect, subscapularis, humeral avulsion of glenohumeral ligament, glenoid avulsion of glenoid humeral ligament, inferior recess, bare area of humerus, and rotator cuff. The posterior portal is utilized for introducing the jig.

Then, an anteroinferior portal is made using an outside‐in technique in the rotator interval using a spinal needle. In the same direction of the needle, use a No. 11 blade to make a skin incision of size 3 cm, piercing the deep fascia, so that index finger of the hand enters the anteroinferior portal and able to touch the anterior glenoid, and insert an 8 mm cannula in the anteroinferior portal; this portal is now the working portal (Figure 4b). The coracohumeral ligament has to be released to aid in easy graft passage later (Figure 4c).

An anterosuperior portal for viewing portal is made in the rotator interval or through the trans cuff on the side of the biceps. Now, switch the scope to the viewing portal from the posterior portal. Then, remplissage suture anchors are placed in the Hill‐Sachs defect at the first point of contact during dislocation. Then, the sutures are used to help in the traction of the head during the passage of the bone graft.

### Elevation and Retraction of the Labrum

**FIGURE 5:**
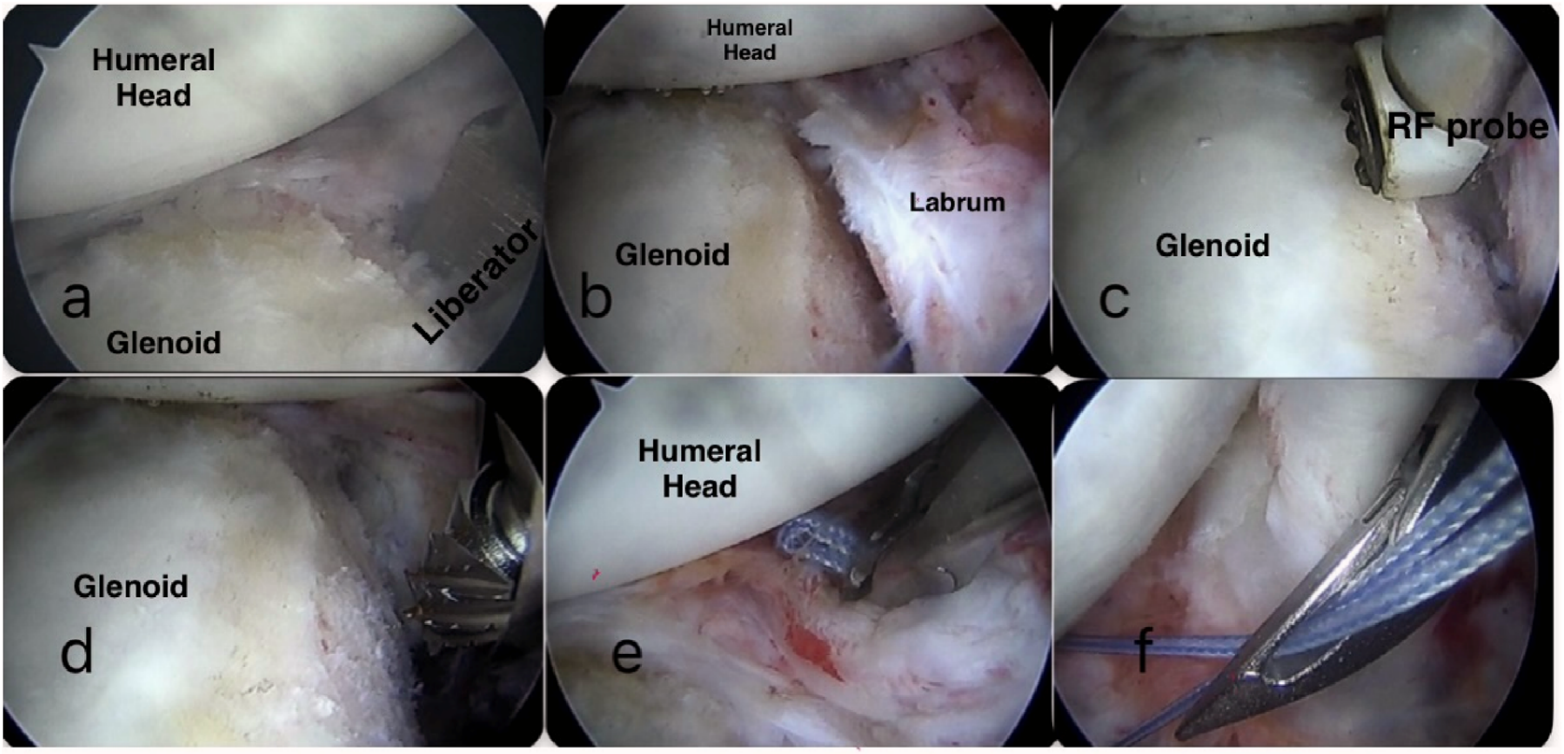
Arthroscopic preparation of the anterior glenoid viewed from the anterosuperior viewing portal in a left shoulder. (a) Use of a periosteal labral liberator to delineate the plane between the glenoid and labrum. (b) Progressive mobilization until the labrum “floats” above the glenoid surface. (c) Electrocautery used to expose raw bone along the anterior glenoid rim from the 1 to 6 o'clock position. (d) Burr preparation of the glenoid bed to expose cancellous bone. (e) Passage of the critical inferior labral suture (“most important bite”) between the 5:30 and 6 o'clock position, taken at least 1 cm from the labral margin. (f) Retrieval of the suture using a parrot‐beak device through the trans‐subscapularis portal.

For ideal viewing of the labrum and liberation of the labrum, an anterosuperior viewing portal is necessary, and we get a bird's eye view of the entire glenoid. Using the labral liberator, identify the extent of the labral tear and the place of the labral tear. Using the labral liberator, identify the plane between the labrum and glenoid and gently tap on the liberator, until the give‐away feeling is felt; the medial liberation of the labrum is essential to get the labral tissue mobile (Figure 5a). After releasing the labrum medially, extend the release of the labrum in the same plane superiorly up to 2 o'clock position and inferiorly up to 6 o'clock position until you reach the normal attached labrum. During superoinferior labral liberation, the plane should be kept the same; if done in different planes, the labrum tissue will be shredded. When the liberation of the labrum is done completely, the labral tissue will float above the level of the glenoid (Figure 5b), and this ensures the labrum is fully mobilized. Use a grasper to grasp the labrum and look for reducibility of the labrum. You must ensure that after liberating the labrum there is a good volume of space to accommodate the bone graft.

### Preparation of the Anterior Glenoid

The anterior glenoid articular edge is prepared by using radio frequency ablation to prepare the bed for labrum tissue to heal (Figure 5c). The anterior glenoid area is prepared using a combination of burr (Figure 5d) and scraping with a periosteal elevator, to expose cancellous bone and to create a uniform bone bed for the graft to sit precisely (Figure 5d).

Labral bite is taken at the inferiormost labral tissue using an antegrade suture passing device—Scorpion (Arthrex, Naples, FL) and Size 0 FiberWire (Arthrex, Naples, FL). The FiberWires are retrieved through the subscapularis muscle using parrot beak; similarly, another bite in the labral tissue at the 3 o'clock position is taken and retrieved through the same subscapularis and parked there with the previous FiberWires. These FiberWires are clamped with artery forceps and used to give traction during graft passage to provide space and prevent labrum tangling.

### Introduction of Jig and Placement of Drill Holes

**FIGURE 6:**
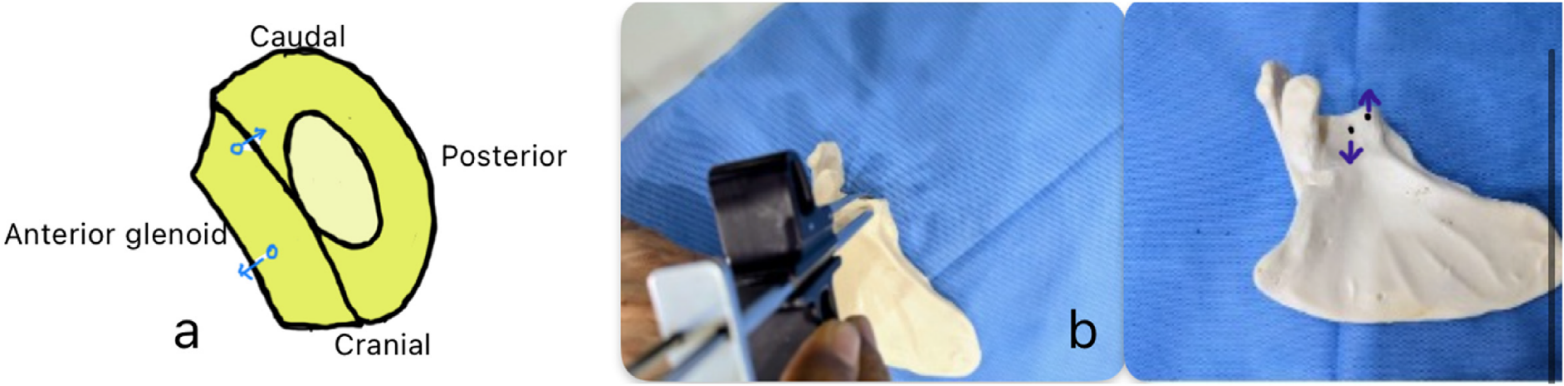
Orientation of the drill guide and resultant glenoid holes. (a) Illustration showing the drill guide holes, with the caudal hole positioned slightly lateral relative to the cranial hole. (b) Lateral inclination of the caudal portion of the graft achieved by controlled tilting of the jig. (c) Final position of the drill holes on the anterior glenoid, facilitating optimal graft seating and fixation.

The glenoid jig is inserted through the posterior portal, with the jig's horizontal arm resting slightly more tilted caudally on the glenoid articular surface and aiming tip hitching on the anterior glenoid towards the defect area, i.e., anteroinferior area. Then, the glenoid is drilled superiorly and inferiorly with the cannulated drill bit (Figure 6a) and, the drill pin cover is removed, ensuring that free flowing of water is ensured that no bone particle obstruction is in the cannulated drill bit. It is to be noted that the caudal drill hole is more towards the glenoid surface, and the cranial hole is far from the glenoid articular surface (Figure 6c).

The nitinol guide wire is inserted through the inferior and superior cannulated drill bit through the posterior portal, and the nitinol wire is retrieved through the working portal. The glenoid jig and cannulated drill bit are removed while keeping the nitinol wire in place without intertangling.

### Graft Passage

**FIGURE 7:**
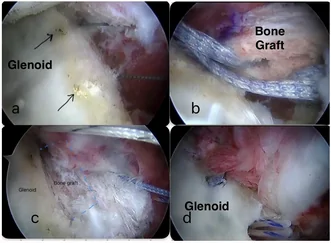
Arthroscopic graft insertion and final labral repair in a left shoulder, viewed from the anterosuperior viewing portal. (a) Electrocautery markings placed on the anterior glenoid to guide anchor placement while avoiding interference with the FiberTape used for graft fixation. (b) Passage of the bone graft into the joint using a Kocher forceps while the labrum is retracted with sutures parked through the subscapularis portal—the most technically demanding step of the procedure. (c) Final graft positioning with the graft seated slightly more proud caudally than cranially, restoring the anterior glenoid contour. (d) Completion of labral advancement and repair over the bone graft, reestablishing the bumper effect and enhancing anterior shoulder stability.

It is the most difficult step in this procedure. FiberTape (Arthrex, Naples, FL) is loaded in the inferior nitinol loop posteriorly and pulled slowly through the working portal (Figure 7b). The FiberTape from the working portal is passed through the inferior hole of the prepared bone graft and looped and exits through the superior hole; the FiberTape is then loaded in the superior nitinol loop in the working portal and pulled through the posterior portal.

The graft is held with Kocher forceps, and the graft is passed through vertical fashion by pulling the inferior FiberTape; the graft is passed through the working portal, and once it reaches the joint space, the graft is made to sit on the anterior glenoid by pulling both ends of the FiberTape. The tensioning is done on the FiberTape using a specialized tensioner (Arthrex, Naples, FL), and tension is applied at 40 N.

### Labral Repair

The labral repair is done by releasing the FiberWire parked at the subscapularis portal. Try to mark the intended anchor position avoiding the nitinol wire in the glenoid tunnels (Figure 7a). The inferior labral bite FiberWire is retrieved through the working portal and fixed in the anterior glenoid edge at the region coinciding with the middle of the graft so as not to damage the graft FiberTape. PushLock (Arthrex, Naples, FL) is used to fix the labrum. Similarly, the other labral FiberWires is retrieved and fixed in the anterior glenoid at 2 o'clock position. If additional labrum needs repair, it is repaired with PushLock as necessary, thereby completing labral repair (Figure 7d).

## DISCUSSION

The arthroscopic iliac crest bone block procedure offers a powerful solution for glenoid bone loss in shoulder instability, with several advantages over the open procedures. First, being minimally invasive, arthroscopy allows for minimal soft tissue damage; this in turn may be correlated with less pain postoperatively, quicker rehabilitation, and superior cosmesis.^4^ Furthermore, the arthroscopic visualization of the entire joint permits a full assessment of concomitant pathologies, such as labral tears, rotator cuff status, and articular cartilage damage, which may all be dealt with at the same time.

When certain open bone block procedures call for wide dissection with consequent potential for increased morbidity, including subscapularis damage, the advantage of the arthroscopic method lies in its mitigation of some or all of this morbidity. Open approaches have provided good clinical results, but with better visualization and instrumentation, arthroscopy will provide the same level of good results with less trauma to the soft tissues.^5^, ^6^

In an arthroscopic bone block versus Latarjet setting, both open and arthroscopic, there are certain distinct considerations. The Latarjet operation, wherein the coracoid process with its attached conjoined tendon is transferred anteriorly to the glenoid, transferred conjoined tendon offers a “sling effect,” thereby providing dynamic stability. However, the potential complications of the Latarjet procedure include nonunion of the coracoid transfer, neurovascular injury, and stiffness.^7^ In contrast, the iliac crest bone block procedure restores the native glenoid anatomy by augmenting the articular surface with autologous bone, which biologically incorporates to the native bone, thus does not altering the coracoglenoid biomechanics.^6^ The choice between these procedures is largely made on surgeon preference, extent and type of bone loss, and patient factors.

Using an autologous iliac crest graft is one strong point of this technical procedure. Autografts serve as the gold standard for bone grafting, bearing osteoinductive, osteoconductive, and osteogenic qualities; hence, a reliable bone union is formed that fuses with the native glenoid.^8^ Biological incorporation is of utmost importance for long‐term stability and for preventing resorption of the graft. This technique emphasizes carefully preparing and positioning the graft such that a large surface area contacts the prepared glenoid bed to favor healing.

The preparation of the anterior glenoid articular edge is a very important technical point. Ensuring a parallel bone bed with the burr and periosteal elevator for maximum graft‐host contact is of utmost importance in respect to graft healing and stability. Studies have shown the importance of precise medial‐lateral positioning of the bone block, since grafts placed too medially are associated with increased bone resorption and do not prevent instavility.^8^ Likewise, full labral liberation and repair must be performed. The release of the labrum, beginning superiorly and going inferiorly until it floats, warrants its full free mobilization, thus allowing the reduction of labrum over the bone block and increasing the repair's stability. Another valuable technical trick is the usage of FiberWires to retract the labrum during the passage of the graft, preventing entanglement and allowing smooth insertion of the bone block.

This graft‐passage method with FiberTape loaded through nitinol loops offers a controlled and efficient method of inserting the bone block into the joint and accurately positioning it. A special tensioner has been used to apply an exact tension of 40 N on the FiberTape, ensuring that the fixation is secure enough to prevent the graft from shifting at the glenoid.

Comparison with graft fixation strategies such as screw fixation, cerclage tape, and suspensory fixation have shown satisfactory fixation strength and clinical outcomes, while potentially reducing the risks of graft fracture and hardware irritation.^9^, ^10^, ^11^

Postsurgical management and rehabilitation are critical steps toward success in this procedure. Immobilization is necessary in the initial 3 weeks, followed by a targeted physical therapy program aimed at enhancing range of motion, strengthening, and gradual reengagement in activities. This is important to achieve functional optimization and reduce recurrence.

The advantages, limitations, and key technical points of this technique are summarized in Tables 1 and 2.

**TABLE 1:** Advantages & Disadvantages

Advantages	Disadvantages
Restores the native glenoid anatomy	Risk of graft malposition if jig alignment is inaccurate
Smaller incisions, thereby less postoperative pain and better cosmesis	Technically demanding procedure with a steep learning curve
No hardware‐related complications when FiberTape is used for graft fixation	Requires specific instrumentation and implants
Avoids coracoid transfer and preserves the coracoacromial arch	Long‐term outcome data are still limited when compared with Latarjet procedure
Simultaneous treatment of associated pathology (labral tear, Hill‐Sachs lesion)	Increased operative time compared with open procedure
Iliac crest autograft provides reliable biological incorporation	Increased financial cost due to implants
Exteriorize the bone graft hence less chance of secondary degeneration	

**TABLE 2:** Pearls & Pitfalls

Pearls	Pitfalls
Preoperative 3D CT to evaluate the glenoid bone loss	Underestimating the glenoid defect can lead to undersized graft size
Proper patient positioning in lateral decubitus to facilitate graft harvesting and arthroscopic visualization	Suboptimal positioning making graft harvesting and graft passage difficult
Complete medial and circumferential labral liberation until the labrum floats	Incomplete labrum liberation cause difficulty in graft passage and subsequent labral repair
Preparing a flat, bleeding cancellous glenoid bed for optimal graft incorporation	Inadequate glenoid preparation results in poor graft contact and healing
Using traction sutures on the labrum to prevent entanglement during graft passage	Labrum getting trapped between graft and glenoid
Ensuring graft is positioned flush to the native glenoid surface	Medial or lateral placement of graft can result in risk of graft resorption or arthritis
Controlled tensioning of FiberTape fixation to achieve stable graft compression	Excessive tension causing graft fracture or insufficient stability
Confirming graft stability and labral repair arthroscopically before closure	Failure to reassess graft alignment and labral repair may result in poor outcomes
Partial release of coracoid part of coracoacromial ligament	Inadequate release causing resistance during graft delivery

3D, 3‐dimensional; CT, computed tomography.

The major complications that can be encountered include graft malposition, nonunion, or resorption; neurovascular injury; infection; and recurrent instability. Preoperative considerations, including surgical technique and even patient selection, are keys in minimizing the risks involved. The anatomical landmarks and portal placement described herein are essential for safely and effectively performing this operation, especially regarding the outside‐in technique for the anteroinferior working portal. Although early results of arthroscopic fixation bone block procedures using cerclage tape are promising, additional studies with longer‐term follow‐up are required to establish these methods in larger patient groups.^12^

In conclusion, the arthroscopic bone block procedure described here, using iliac crest graft, is a technically demanding yet simple method for addressing glenoid bone loss in shoulder instability. Combining careful surgical steps with advantages of arthroscopy and autologous grafting, this technique attempts to restore glenoid anatomy to provide a stable buttress and ultimately improve patient outcomes in difficult cases of recurrent glenohumeral instability.

## DISCLOSURES

The authors (K.N.S., A.R., N.R., J.N.) declare that they have no known competing financial interests or personal relationships that could have appeared to influence the work reported in this article.
